# Utilization of Emergency Service of Obstetrics and Gynecology: A Cross-Sectional Analysis of a Training Hospital

**DOI:** 10.14740/jocmr2013w

**Published:** 2014-11-19

**Authors:** Huseyin Aksoy, Ulku Aksoy, Mustafa Ozturk, Sezin Ozyurt, Gokhan Acmaz, Ozge Idem Karadag, Burak Yucel, Turgut Aydin

**Affiliations:** a Department of Obstetrics and Gynecology, Kayseri Military Hospital, Kayseri, Turkey; b Department of Obstetrics and Gynecology, Kayseri Memorial Hospital, Kayseri, Turkey; c Department of Obstetrics and Gynecology, Etimesgut Military Hospital, Ankara, Turkey; d Department of Obstetrics and Gynecology, Kayseri Education and Research Hospital of Medicine, Kayseri, Turkey; e Department of Obstetrics and Gynecology, Kayseri Acibadem Hospital, Kayseri, Turkey

**Keywords:** Emergency, Obstetrics, Gynecology

## Abstract

**Background:**

Overutilization and inappropriate use of emergency departments (EDs) by patients with non-urgent health problems has become a major concern worldwide. This study aims to describe the characteristics of obstetric and gynecologic patients admitted to the Department of Emergency Obstetric and Gynecology.

**Methods:**

A retrospective and cross-sectional study was designed at our Emergency Service of Obstetrics and Gynecology of Kayseri Education and Research Hospital of Medicine between January 1 and December 31, 2013. A total of 30,853 patients applying to emergency service were retrospectively analyzed from the admission charts, patient files and hospital automation system. Patients were assessed in terms of demographic features, presentation times, complaints, admission type (with own facilities, with consultation or with ambulance), diagnoses (urgent or non-ergent), discharge rates, clinical admission, rejection rate of examination, and rejection rate of hospitalization.

**Results:**

A total of 30,853 patients were analyzed retrospectively. The mean age of patients was 27.69 ± 8.44 years; 51% of patients were between 20 and 29 years old. The categories of patients in urgent and non-urgent were 69% and 31% respectively. Most common presentation time period was between 19:00 and 21:00. Labor pain, pain and bleeding during pregnency, routine antenatal control, pelvic pain and menstrual irregularity were the most common complaints. Labor pain with the rate of 21% was the most common cause of ED admission. All patients who presented with labor pain were hospitalized. Patients hospitalized for labor constituted 56% of all hospitalized patients. Among patients, 62% were treated on an outpatient basis and 38% were hospitalized. Of patients, 3.54% refused to hospitalization. The cases using the ambulance to admission constituted 1.07% of all ED patients. Of these patients who have used ambulance 3.65% refused to the patient examination.

**Conclusions:**

To improve the obstetric and gynecologic emergency medical care in Turkey, demographic properties and other characteristics of patients should be analyzed in detail. Detailed analysis of the data contributes to the further design and perspective of the EDs.

## Introduction

Emergency department (ED) use continues to rise in Turkey, and worsening crowding compromises patient care [1]. The need to prioritize these patients is stressed by the considerable demand for emergency care, frequent ED overcrowding and limited resources. EDs are designed to provide “rapid, high-quality, continuously accessible, unscheduled care” for a wide range of acute illnesses and injuries [2].

An important portion of patients who attend hospital EDs presenting with health problems is classified as non-urgent. With many EDs experiencing long waits and overcrowding, it has been suggested that providing primary care services in EDs for patients with non-urgent problems may be an efficient and cost-effective alternative to emergency care. But EDs often evaluate obstetrical and gyecological problems as urgent. The management of obstetrical and gynecological emergencies is directed towards preservation of maternal and fetal life, health and function, in particular the conservation of sexual function and the perpetuation of fertility if possible. There is no population-based information from Turkey to document the characteristics of those who use the ED compared with those who do not and whether the principles of Turkey universal health insurance hold true for obstetric and gynecology patients. To provide high-quality emergency medical care, we need to assess the management of patients through precise patient data documentation and data collecting systems.

This study aims to describe the characteristics of obstetric and gynecologic patients admitted to the Kayseri Education and Research Hospital of Medicine, Department of Emergency Obstetrics and Gynecology. In this paper, we present a descriptive analysis of attempted solutions and outcomes, so that others might adapt successful approaches as demands increase and resources diminish.

## Materials and Methods

We carried out a retrospective study of the ED utilization at Obstetrics and Gynecology Emergency Service of an Education and Research Hospital, a tertiary referral center in Turkey. Our study was approved by the Local Ethics Committee of Erciyes University Faculty of Medicine. Medical records of 30,853 patients who applied to ED between January 1, 2013 and December 31, 2013 were examined. Data of this retrospective and cross-sectional study were retrieved from patient files and automation system. Patients were assessed in terms of demographic features, presentation times, complaints, apllication type (with own facilities, with consulation or with ambulance), diagnoses (urgent or non-urgent), discharge rates, clinical admission, rejection rate of examination and rejection rate of hospitalization. Patients whose medical records could not be obtained were excluded. The staff of the ED comprises registered obstetricians and gynecologists.

## Results

**Figure 1:**
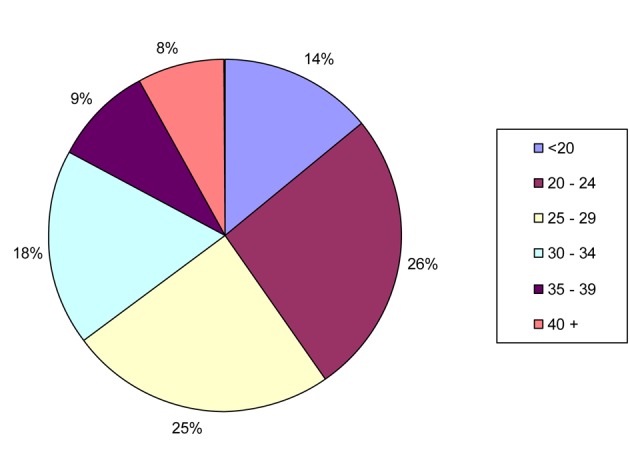
Age distribution of patients.

**Figure 2:**
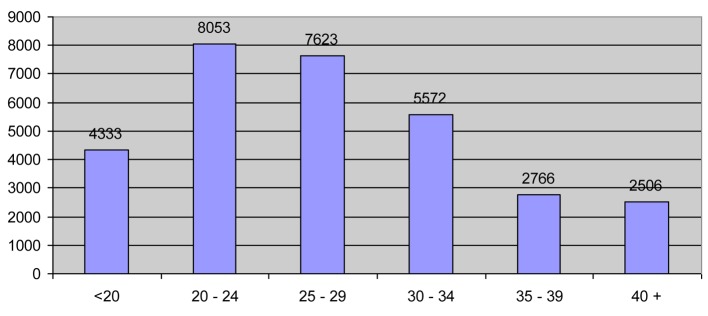
Frequency of application by age.

A total of 30,853 patients were analyzed. The mean age of patients was 27.69 ± 8.44 years; 51% of patients were between 20 and 29 years old. The distribution of age and the frequency of application by age were illustrated in Figures 1 and 2. The categories of patient in urgent and non-urgent were 69% and 31%, respectively (Emergency patient/Non-emergency patient ratio = 42%). The distributions of patients in terms of urgent and non-urgent indications were summarized in Tables 1 and 2.

**Table 1:** Distribution of Urgent Indications

Urgent indications	Outpatient number (n)	Outpatient percent (%)	Inpatient number (n)	Inpatient percent (%)
Labor pain	0	0.00	6,478	21.00
Threatened abortion	1,186	3.84	1,841	5.97
Complet/incomplet abortion	51	0.17	349	1.13
Hyperemesis gravidarum	753	2.44	317	1.03
Abdominal pain (first trimester)	540	1.75	27	0.09
Abdominal pain (second trimester)	1,014	3.29	96	0.31
Abdominal pain (third trimester)	1,203	3.90	156	0.51
Abnormal uterine bleeding	612	1.98	372	1.21
Pelvic pain	1,307	4.24	61	0.20
Pregnancy and vomiting	102	0.33	0	0.00
Risk of premature birth	0	0.00	978	3.17
Pregnancy and urinary tract infection (first trimester)	156	0.51	7	0.02
Pregnancy and urinary tract infection (second trimester)	264	0.86	9	0.03
Pregnancy and urinary tract infection (third trimester)	426	1.38	37	0.12
Urinary tract infection	534	1.73	5	0.02
Decrease in fetal movement	235	0.76	71	0.23
Ectopic pregnancy	132	0.43	222	0.72
Pregnancy and shortness of breath	47	0.15	0	0.00
Pregnancy and traffic accident	22	0.07	7	0.02
Pregnancy and falling	183	0.59	41	0.13
Ovarian cyst	319	1.03	94	0.30
Pelvic inflamatory disease	135	0.44	63	0.20
Vulvar edema	74	0.24	0	0.00
Vulvadynia	87	0.28	0	0.00
Postoperative complications	46	0.15	69	0.22
Mastitis	135	0.44	0	0.00
Birth at home	0	0.00	55	0.18
Postpartum pain	64	0.21	0	0.00
Early membrane rupture	0	0.00	151	0.49
Postcoital bleeding	43	0.14	32	0.10
Lost intra uterine device	51	0.17	0	0.00
Hypertention in pregnancy	42	0.14	39	0.13
Placenta previa	12	0.04	14	0.05
Total	9,775	31.68	11,591	37.57

**Table 2:** Distribution of Non-Urgent Indications

Non-urgent indications	Outpatient number (n)	Outpatient percent (%)	Inpatient number (n)	Inpatient percent (%)
Antenatal control	3,017	9.78	0	0.00
Irregular menstruation	738	2.39	0	0.00
Dismenore	636	2.06	0	0.00
Delay in menses	816	2.64	0	0.00
IUD control	366	1.19	0	0.00
Postoperative control	696	2.26	0	0.00
Vaginitis	852	2.76	0	0.00
Suspected pregnancy	780	2.53	0	0.00
Pregnancy and enteritis	264	0.86	0	0.00
Pregnancy and headache	151	0.49	0	0.00
Pregnancy and weakness	113	0.37	0	0.00
Pregnancy and vertigo	127	0.41	0	0.00
Pregnancy and common cold	297	0.96	0	0.00
Pregnancy and epistacsis	19	0.06	0	0.00
Pregnancy and teeth pain	97	0.31	0	0.00
Pregnancy and insect bite	23	0.07	0	0.00
Contraception	71	0.23	0	0.00
Condilom	13	0.04	0	0.00
Pregnancy and itcing	68	0.22	0	0.00
Constipation	58	0.19	0	0.00
Postpartum control	43	0.14	0	0.00
Infertility	51	0.17	0	0.00
Hymenal control	34	0.11	0	0.00
Anemia	87	0.28	0	0.00
Urinary incontinence	8	0.03	0	0.00
Pregnancy and neck pain	17	0.06	0	0.00
Primary amenore	4	0.01	0	0.00
Puberte precox	3	0.01	0	0.00
Preoperative control	38	0.12	0	0.00
Total	9,487	30.75	0	0.00

**Figure 3:**
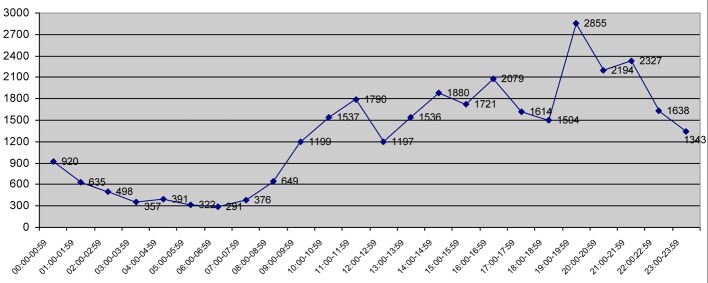
Distribution of application hours of patients.

Most common presentation time period was between 19:00 and 21:00. The distribution of application hours of patients was shown in Figure 3. Labor pain, pain and bleeding during pregnency, routine antenatal control, pelvic pain and menstrual irregularity were the most common complaints. Labor pain with the rate of 21% was the most common cause of ED admission. All patients with labor pain were hospitalized. Patients hospitalized for labor constituted 56% of all hospitalized patients. Among patients, 62% were treated on an outpatient basis and 38% were hospitalized. Of patients, 3.54% refused to hospitalization. The cases using the ambulance to admission constituted 1.07% of all ED patients. Of these patients who have used ambulance 3.65% refused to the patient examination.

## Discussion

The EDs are the source of acute care for most critically ill or injured patients. Hospital EDs are designed to provide “rapid, high-quality, continuously accessible, unscheduled care” for a wide range of acute illnesses and injuries. Turkey’s emergengy health system differs from that of the many other countries due to the universality of care (free access). In Turkey, there is a National Health System (NHS), which provides universal and free health coverage including emergency health care. All people in Turkey, regardless of origin and legal status, have the right to emergency care. This legislation guaranteed access to care for all patients presenting to the ED, regardless of their ability to pay. It has been estimated that more than the population use EDs at least once every year.

An important portion of patients who attend hospital EDs present with health problems that are classified as non-urgent. Inappropriate ED use for non-urgent problems has been suggested as a probable primary contributor to ED overcrowding. The inappropriate use of ED is a major health problem, especially in countries with publicly funded health systems such as Turkey. According to the 2010 statistical yearbook of the Ministry of Health of Turkey, 302 million people applied to private and public hospitals [3]. In the same year, a total of approximately 50 million patients were admitted to hospitals in Istanbul, which is the most crowded city in Turkey [4], and approximately 10 million of them were admitted directly to ED [5]. When compared by population, our country has one of the highest ED use. Although an increasing number of visits to hospital EDs has been described in Turkey, a decreasing proportion of these visits results in a outpatient admission [6]. The use of the ED as a source of primary care for many patients has been suspected as a cause of this finding. The inappropriate use of EDs makes it difficult to guarantee access for real emergency cases, decreases the readiness for care, produces negative spillover effects on the quality of emergency services, and raises overall costs.

The proportion of non-urgent visits in our country has been estimated to be between 28% and 76% in a pediatric ED [7], but we have no data available for adults. In Europe, values around 40% have been reported [8]. The percentage of patients going to an ED for non-urgent problems is between 9% and 54% in the USA [9]. We found this rate for non-urgent visits 31% in obstetric and gynecology ED. It is smiliar to European countries. The national emergency health care system in Turkey is characterized by the universality of care (free access), a hierarchical structure with three levels of increasing complexity (primary, secondary and tertiary levels), and an integrated approach to delivering care for all types of health needs. Kayeri is a big size city located in Central Anatolia, with 1,295,355 inhabitants. The city has a public health system including 50 primary health care centers (PHC) spread across the city, each staffed (at minimum) by a general physician, a nurse and a receptionist. The secondary level of care comprises specialist physicians, who work in ambulatory clinics, while the tertiary level of care comprises four hospitals [10].

We found that among urgent patients, 45% were treated on an outpatient basis and 55% were hospitalized. Labor pain with the rate of 21% was the most common cause of ED admission in urgent group. Antenatal control was the most common cause of ED in non-urgent group (9.8%). Of patients who used ambulance, 3.65% refused emergency examination. This paradox demonstrates that most of the patients do not feel themselves as real urgent patients. Roughly half of the patients who applied to ED were between 20 and 29 years old, due to the fact that obstetric population included women in childbearing age. This makes our study differnt from the other studies including the patients with older ages who apply to general EDs. As summarized in Figure 3, most of the patients applied to our ED between 17:00 and 23:00. We thought that this might be attributable to social factors of the patients like their husbands’ coming home hours.

In obstetrics, there are two patients to care for instead of one, a mother and a baby. The management of one patient heavily affects the management of the other. Sometimes the decision has to be made to care for one patient at the expense of the other, i.e., care for the mother first. The second patient (the baby) may be viable or not. Antenatal control (non-urgent group) patients have several factors to choose emergency services instead of primary and specialized health services: the desire to receive care on the same day, the possibility of being attended to in a setting where it is possible to do laboratory and other tests, and the belief that emergency room services are able to solve complex types of health problems.

To improve the obstetric and gynecologic emergency medical care in Turkey, demographic properties and other characteristics of patients should be analyzed in detail. Detailed analysis of the data contributes to the further design and perspective of the EDs.
